# Benefit of achieving lactate clearance versus central venous oxygen saturation target as microcirculation end point resuscitation in severe sepsis and septic shock

**DOI:** 10.1186/cc14021

**Published:** 2014-12-03

**Authors:** R Sinto, S Suwarto, KC Lie, D Widodo, HT Pohan

**Affiliations:** 1 Division of Tropical and Infectious Disease, Department of Internal Medicine, Faculty of Medicine Universitas Indonesia, Jakarta, Indonesia

## Introduction

In severe sepsis and septic shock patients, lactate clearance >10% and central venous oxygen saturation (ScvO_2_) >70% are accepted parameters of tissue oxygenation adequacy. There is controversy of which parameters better associate with early mortality, and thus should be implemented as the microcirculation end point resuscitation [1-3]. This study was aimed to address the association of achieving either one or two targets of microcirculatory end point resuscitation and early mortality in severe sepsis and septic shock patients.

## Methods

A retrospective cohort study was conducted in severe sepsis and septic shock patients (aged 18 years and older) hospitalized in the ICU, Cipto Mangunkusumo Hospital, Indonesia. Patients' early outcomes were observed during first 120 hours of hospitalization. Cox's regression analysis was used to analyse risk of early mortality in subject groups achieving lactate clearance target only, ScvO_2 _target only, both targets, and not achieving any target in 6 hours after onset of resuscitation.

## Results

**Figure 1:**
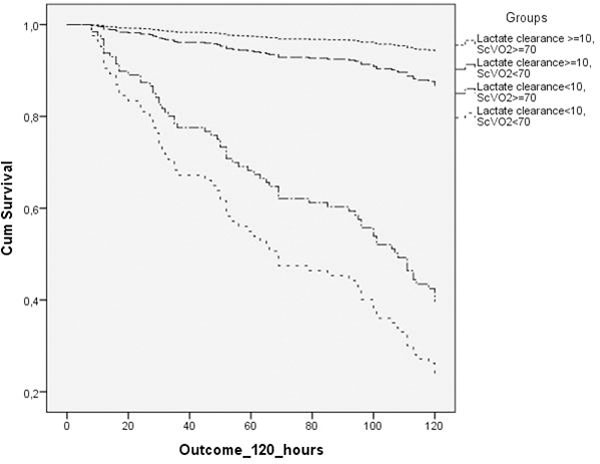
**Survival analysis of groups based on target achievement**.

Subjects consisted of 268 patients. Early mortality developed in 70 subjects. Fifty-four subjects achieved lactate clearance target only, 16 achieved ScvO_2 _target only, 138 achieved both targets, 60 did not achieve any target. Subjects who achieved both targets had a significant lowest early mortality risk (*P *= 0.104 compared with subjects achieved lactate clearance target only and *P *= 0.000 compared with remaining subject groups) (Figure 1). In subgroup analysis of subjects who achieved lactate clearance or ScvO_2 _target only, failure to achieve lactate clearance target associated with higher early mortality risk (hazard ratio 5.92; 95% CI 2.18 to 16.01).

## Conclusion

Achieving both lactate clearance and ScvO_2 _targets in 6 hours after onset of resuscitation associates with lowest early mortality risk in severe sepsis and septic shock patients. Patients who achieve lactate clearance target only have a significant lower early mortality risk compared with those who achieve ScvO_2 _target only.
